# Supplementary material for: Fatal opioid overdoses during and shortly after hospital admissions in England: A case-crossover study
Source: PLoS Med. 2021 Oct 5;18(10):e1003759. doi: 10.1371/journal.pmed.1003759 (PMC8491890; doi:10.1371/journal.pmed.1003759)
Supplement: S1 Appendix — Table A: Distribution of ICD-10 diagnoses in opioid-related deaths in England between January 1, 2010 and December 31, 2019. Table B: Results of alternative self-controlled methodologies. Values are conditional odds ratios of opioid-related deaths (95% CIs). Table C: Results of case-crossover analysis stratified by sex and calendar year of death. Values are conditional odds ratio of opioid-related death (95% CIs). Table D: STROBE Checklist. Fig A: Flowchart showing how the exposure status on the day of death was determined. Fig B: Distribution of age at death for 13,609 people who died due to fatal opioid overdose in England between January 1, 2010 and December 31, 2019. Deaths at ages under 18 or over 65 were excluded from the study. Fig C: Number of hospital admissions in the 730 days prior to death among 13,609 people who died due to opioid overdose in England between January 1, 2010 and December 31, 2019, by 10-day period. Fig D: Results of alternative self-controlled methodologies. The chart shows conditional odds ratios with 95% CIs. Fig E: Results of case-crossover analysis stratified by sex and calendar year of death. Values are conditional odds ratio of opioid-related death (95% CIs). ICD-10, International Classification of Diseases-10th Revision; STROBE, Strengthening the Reporting of Observational Studies in Epidemiology. (PDF) [file pmed.1003759.s001.pdf]

Supplementary Information

S1. Summary of ICD-10 diagnoses recorded in opioid-related deaths 2

S2. Determination of exposure status for deaths 3

S3. Histogram of age of participants at death 4

S4. Density of hospital admission prior to death 5

S5. Results of planned analysis 6

S6. Results stratified by sex and calendar year 8

S7. STROBE checklist 10

S8. References for supplementary information 13

S1. Summary of ICD-10 diagnoses recorded in opioid-related deaths

Table A: distribution of ICD-10 diagnoses in opioid-related deaths in England between 1 January 2010 and 31 December 2019

		Opioids recorded as contributing causes of death. All deaths apart from those with an underlying cause of F11 have at least one of these codes* (% of row)				
ICD-10 code of underlying cause of death	Number of deaths (% of column)	T40.1 Heroin	T40.2 Other opioids	T40.3 Methadone	T40.4 Other synthetic narcotics	T40.6 Unspecified narcotics
Total	13,609 (100.0)	3,953 (29.0)	5,599 (41.1)	3,137 (23.1)	1,834 (13.5)	1,082 (8.0)
X42: accidental poisoning by narcotics and psychodysleptics	10,190 (74.9)	3,549 (34.8)	3,879 (38.1)	2,521 (24.7)	1,107 (10.9)	882 (8.7)
X62: intentional self-poisoning by narcotics and psychodysleptics	1,006 (7.4)	75 (7.5)	619 (61.5)	63 (6.3)	291 (28.9)	56 (5.6)
X44: accidental poisoning by other or unspecified drugs	860 (6.3)	163 (19.0)	371 (43.1)	345 (40.1)	140 (16.3)	53 (6.2)
Y12: poisoning by narcotics and psychodysleptics, undetermined intent	831 (6.1)	121 (14.6)	439 (52.8)	116 (14.0)	179 (21.5)	61 (7.3)
F11: mental and behavioural disorders due to use of opioids	288 (2.1)	24 (8.3)	17 (5.9)	24 (8.3)	1-4**	10-14**
X64: intentional self-poisoning by other or unspecified drugs	194 (1.4)	1-4**	130 (67.0)	15-19**	57 (29.4)	5-9**
Y14: poisoning by other or unspecified drugs, undetermined intent	140 (1.0)	5-9**	97 (69.3)	25 (17.9)	38 (27.1)	1-4**
X41: accidental poisoning by antiepileptic, sedative-hypnotic, antiparkinsonism and psychotropic drugs	53 (0.4)	5-9**	18 (34.0)	20 (37.7)	12 (22.6)	5-9**
Other underlying cause of death	47 (0.3)	5-9**	29 (61.7)	5-9**	5-9**	1-4**

* The case definition also included deaths where T40.0 (poisoning by opium) was recorded, but no deaths with this code were found.

** Censored to prevent disclosure of small cell counts.

S2. Determination of exposure status for deaths

Figure A: Flowchart showing how the exposure status on the day of death was determined

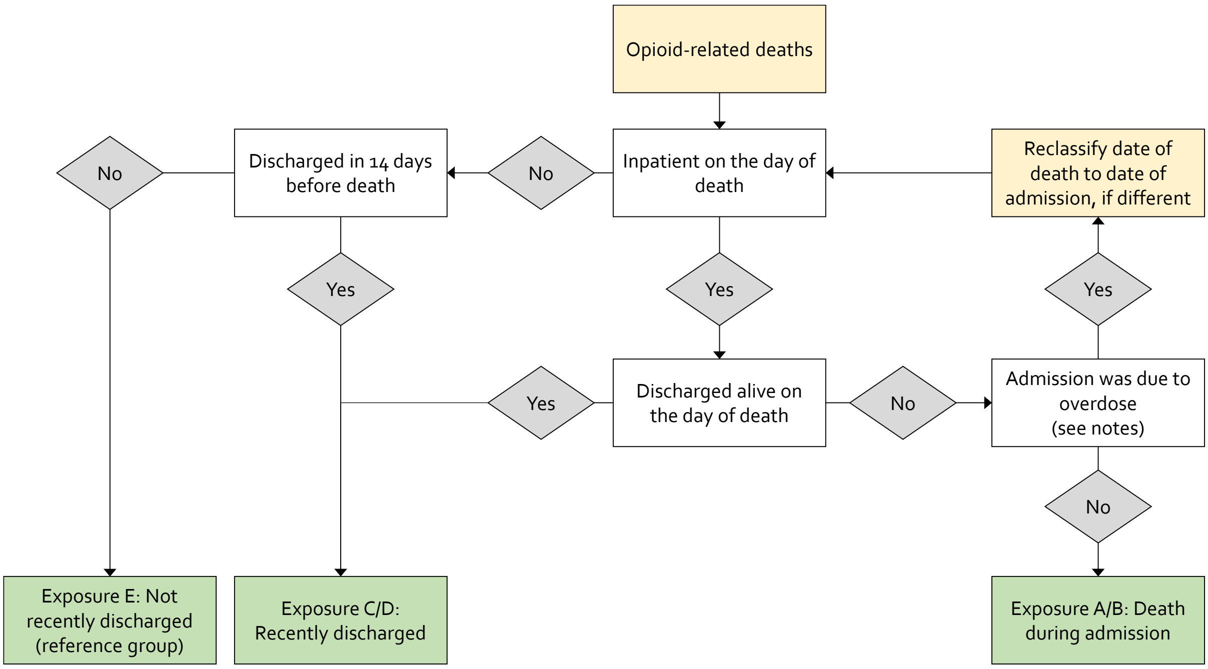

Notes:

Among admissions ending in opioid-related death, admissions due to opioid use identified as those with:

- A drug-related primary diagnosis: ICD-10 X40-44, X60-64, X85, or Y10-Y14 recorded as the primary reason for admission.

- An opioid in any diagnostic position: T40.0-T40.4 or T40.6.

- A primary diagnosis of a known complication of opioid overdose. Consistent with a previous study,[1] we defined the complications as: encephalopathy (G92, G93.1, G93.2); ARDS (J80); respiratory failure (J96.0, J96.9); pulmonary haemorrhage (R04.8); aspiration pneumonia (J69.0); cardiac arrest (I46); ventricular arrhythmia (I47.0, I47.2, I49.0); heart failure (I50); rhabdomyolysis (M62.8, T79.6); paraplegia or tetraplegia (G82); acute renal failure (N17); and intentional self-harm (X60-X84).

S3. Histogram of age of participants at death

Figure B: distribution of age at death for 13,609 people who died due to fatal opioid overdose in England between 1 Jan 2010 and 31 December 2019. Deaths at ages under 18 or over 65 were excluded from the study.

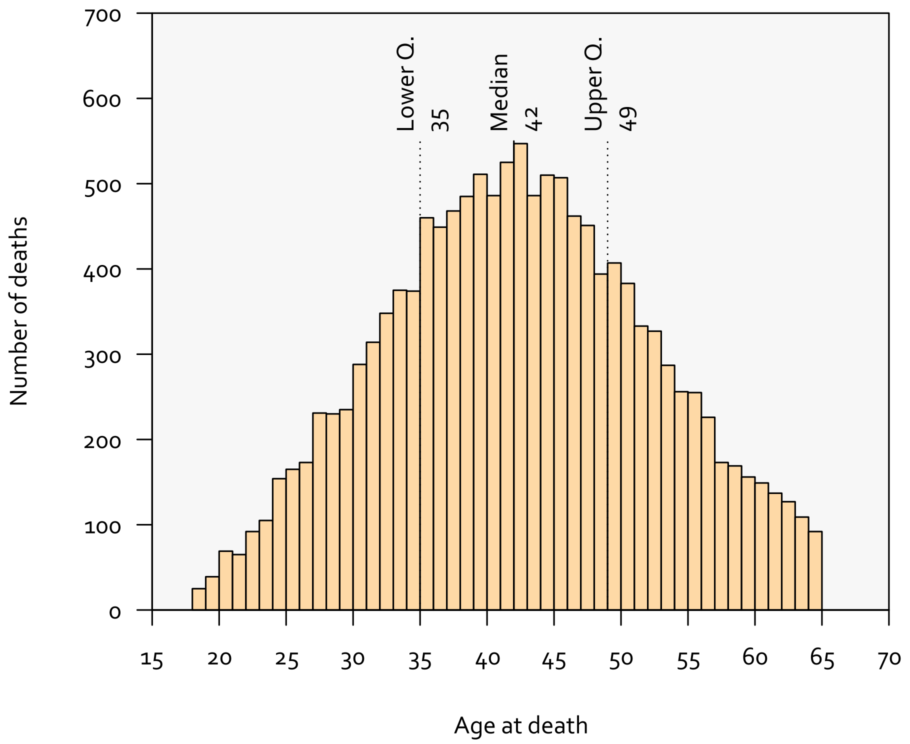

S4. Density of hospital admission prior to death

The number of admissions in the cohort increased during the 730 days prior to death (see figure). This may be related to worsening health or escalating drug use prior to death, secular increases in the rate of hospital admission (in the general population, rates of age-specific hospital admission are increasing [2]), or ageing in the cohort. These factors are likely to explain some of the observed association between hospital admission and death, in addition to the acute effects of the hospital admission. The figure shows that this increase is gradual, which may suggest a limited contribution of these factors to the observed associations.

Figure C: number of hospital admissions in the 730 days prior to death among 13,609 people who died due to opioid overdose in England between 1 Jan 2010 and 31 December 2019, by 10-day period.

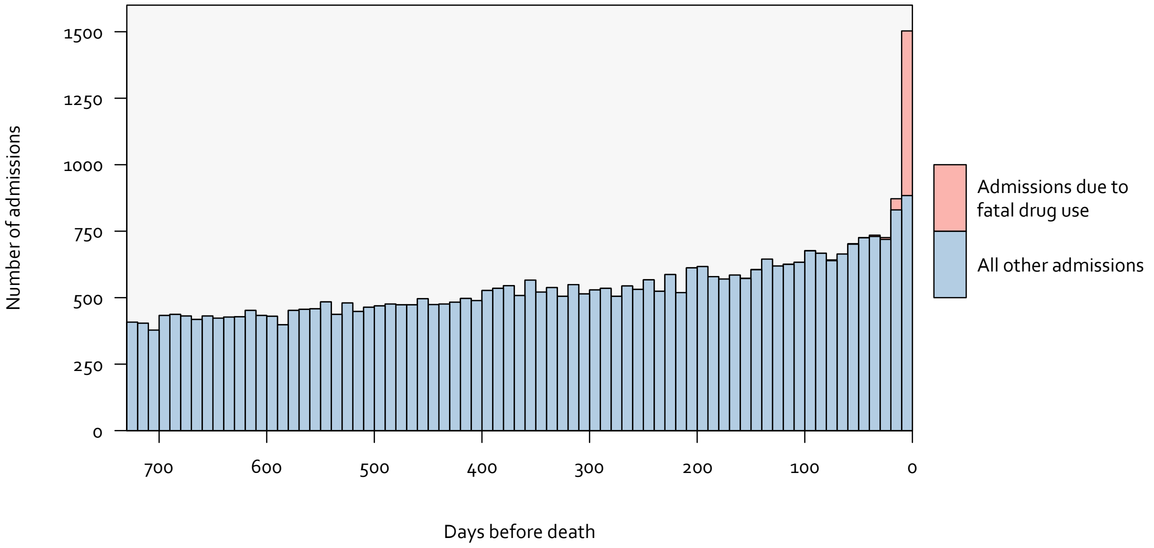

S5. Results of planned analysis

In our protocol [3] we planned to use a self-controlled case series (SCCS) method with observation between 1 January 2010 and 31 December 2019. An assumption of the standard SCCS method is that the observation of exposures (i.e. hospital admissions) is independent of the event.[4] In our study, the event is death and therefore observation of exposures ends at the event. To address this, we planned to use a method for event-dependent exposures designed by Farrington et al,[5] with time-varying age groups of 18-24, 25-34 … 60-64. This method is only suitable for exposures of fixed duration and we were not able to use it for exposure periods A and B (the periods during hospital admission), which vary in duration. This is why we chose to use a case-crossover method in our main analysis. We did fit the model for exposure periods C and D, which are days 1-2 days 3-14 days after discharge and have fixed durations. We did not have sufficient computing resources to fit this model for the whole dataset and therefore used 10 random samples of 500 cases, with a pooled value estimated by fixed-effects meta-analysis, using the R function meta::metagen.[6] Note that this analysis assumes that exposure periods A and B (the hospital admission) have the baseline risk, which is unlikely to affect this analysis substantially because a relatively small proportion of follow-up time was spent in hospital. In addition, we fit models with follow-up ended at death; and with follow-up ended on 31 December 2019 with the time after death treated as exposure-free. Both of these methods were anticipated to be biased, with the model with follow-up ending at 31 December 2019 most strongly overestimating the relative risk associated with the exposure periods.

As a sensitivity analysis, we also repeated the case-crossover analysis with control periods sampled from the periods 365-28 days before death and 1095-28 days before death.

The results of these analyses are summarised the figure and table below. As expected, the SCCS model with follow-up ending on 31 December 2019 showed the largest associations between hospital admission and the fatal opioid overdose. The model for event-dependent exposures produced very similar results to our main analysis. Case-crossover analyses with control windows in the more distant past produced larger associations. Very distant control windows are less likely to represent an ‘exchangeable’ exposure density (i.e. a probability of hospital admission that would be the same in the absence of the event).

Figure D: Results of alternative self-controlled methodologies. The chart shows conditional odds ratios with 95% confidence intervals

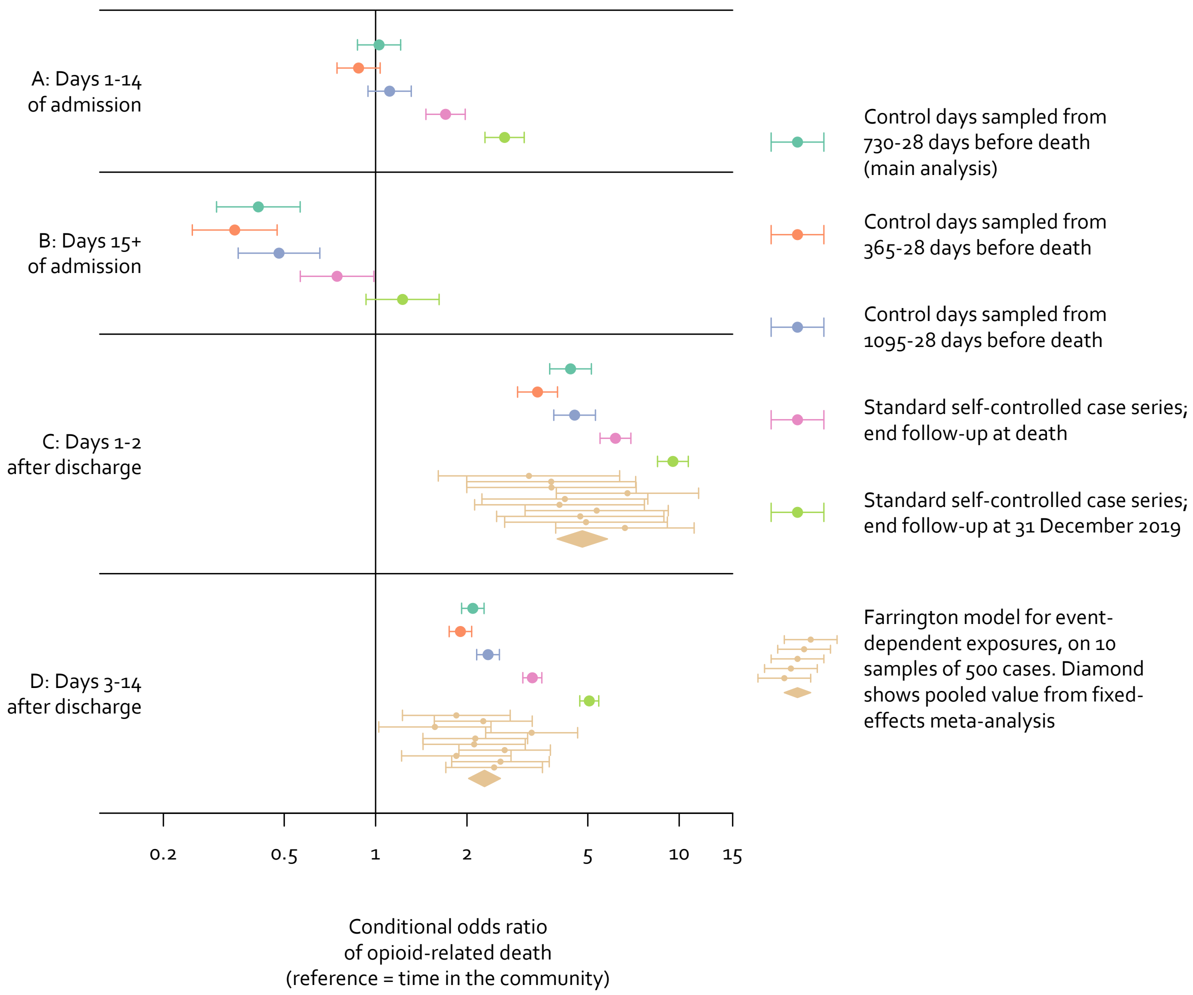

Table B: Results of alternative self-controlled methodologies. Values are conditional odds ratios of opioid-related deaths (95% confidence intervals)

Modelling approach	A: Days 1-14 of admission	B: Days 15+ of admission	C: Days 1-2 after discharge	D: Days 3-14 after discharge
Control days sampled from 730-28 days before death (main analysis)	1.03 (0.87-1.21)	0.41 (0.30-0.56)	4.39 (3.75-5.14)	2.09 (1.92-2.28)
Control days sampled from 365-28 days before death	0.88 (0.75-1.04)	0.34 (0.25-0.47)	3.42 (2.94-3.97)	1.90 (1.75-2.07)
Control days sampled from 1095-28 days before death	1.11 (0.94-1.31)	0.48 (0.35-0.66)	4.52 (3.86-5.30)	2.35 (2.15-2.56)
Standard self-controlled case series; end follow-up at death	1.70 (1.46-1.97)	0.75 (0.56-0.99)	6.17 (5.49-6.93)	3.28 (3.05-3.53)
Standard self-controlled case series; end follow-up at 31 December 2019	2.66 (2.29-3.09)	1.23 (0.93-1.62)	9.53 (8.48-10.72)	5.06 (4.71-5.43)
Farrington model for event-dependent exposures, pooled value from 10 samples of 500 cases	-	-	4.80 (3.96-5.81)	2.28 (2.03-2.58)

S6. Results stratified by sex and calendar year

Figure E: results of case-crossover analysis stratified by sex and calendar year of death. Values are conditional odds ratio of opioid-related death (95% confidence intervals)

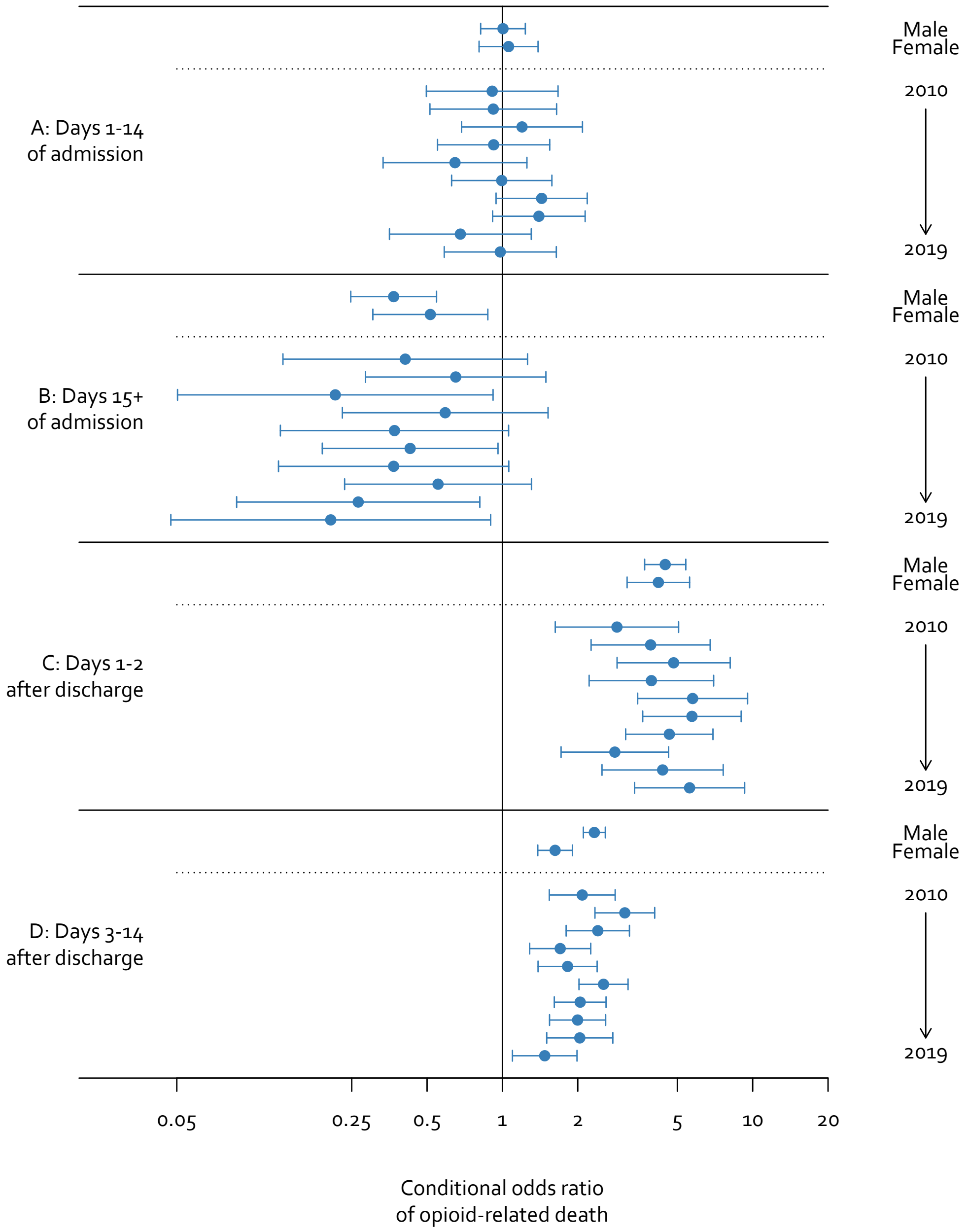

Table C: results of case-crossover analysis stratified by sex and calendar year of death. Values are conditional odds ratio of opioid-related death (95% confidence intervals)

Stratification	Level	A: Days 1-14 of admission	B: Days 15+ of admission	C: Days 1-2 after discharge	D: Days 3-14 after discharge
Sex	Male	1.01 (0.82-1.23)	0.37 (0.25-0.55)	4.47 (3.70-5.40)	2.33 (2.11-2.58)
	Female	1.06 (0.81-1.39)	0.52 (0.30-0.87)	4.20 (3.15-5.60)	1.62 (1.38-1.90)
Calendar year	2010	0.91 (0.50-1.67)	0.41 (0.13-1.26)	2.87 (1.63-5.06)	2.08 (1.54-2.82)
	2011	0.92 (0.51-1.65)	0.65 (0.28-1.49)	3.91 (2.26-6.76)	3.08 (2.34-4.06)
	2012	1.20 (0.69-2.09)	0.21 (0.05-0.92)	4.83 (2.87-8.13)	2.40 (1.80-3.22)
	2013	0.92 (0.55-1.55)	0.59 (0.23-1.52)	3.94 (2.22-6.98)	1.70 (1.28-2.25)
	2014	0.65 (0.33-1.25)	0.37 (0.13-1.06)	5.75 (3.47-9.54)	1.82 (1.39-2.39)
	2015	0.99 (0.63-1.58)	0.43 (0.19-0.96)	5.72 (3.64-8.99)	2.53 (2.02-3.18)
	2016	1.43 (0.94-2.18)	0.37 (0.13-1.06)	4.64 (3.11-6.94)	2.04 (1.61-2.59)
	2017	1.40 (0.91-2.14)	0.55 (0.23-1.31)	2.81 (1.72-4.61)	2.00 (1.54-2.58)
	2018	0.68 (0.35-1.30)	0.27 (0.09-0.81)	4.37 (2.50-7.62)	2.04 (1.50-2.76)
	2019	0.98 (0.59-1.64)	0.21 (0.05-0.90)	5.60 (3.37-9.29)	1.48 (1.10-1.99)

S7. STROBE checklist

Table D: STROBE checklist

	Item No	Recommendation	Page No
Title and abstract	1	(*a*) Indicate the study's design with a commonly used term in the title or the abstract	Title, Abstract ("case-crossover")
		(*b*) Provide in the abstract an informative and balanced summary of what was done and what was found	Abstract
Introduction			
Background/rationale	2	Explain the scientific background and rationale for the investigation being reported	Introduction
Objectives	3	State specific objectives, including any prespecified hypotheses	Introduction ¶3
Methods			
Study design	4	Present key elements of study design early in the paper	Methods ¶1
Setting	5	Describe the setting, locations, and relevant dates, including periods of recruitment, exposure, follow-up, and data collection	Methods/study participants
Participants	6	(*a*) *Cohort study*—Give the eligibility criteria, and the sources and methods of selection of participants. Describe methods of follow-up<br>*Case-control study*—Give the eligibility criteria, and the sources and methods of case ascertainment and control selection. Give the rationale for the choice of cases and controls<br>*Cross-sectional study*—Give the eligibility criteria, and the sources and methods of selection of participants	Methods/study participants; Methods/control days
		(*b*) *Cohort study*—For matched studies, give matching criteria and number of exposed and unexposed<br>*Case-control study*—For matched studies, give matching criteria and the number of controls per case	Methods/control days
Variables	7	Clearly define all outcomes, exposures, predictors, potential confounders, and effect modifiers. Give diagnostic criteria, if applicable	Methods/control days; Methods/exposure status
Data sources/ measurement	8*	For each variable of interest, give sources of data and details of methods of assessment (measurement). Describe comparability of assessment methods if there is more than one group	Methods/exposure status; Supplementary Information/determination of exposure status for deaths
Bias	9	Describe any efforts to address potential sources of bias	Methods ¶1; Methods/control days
Study size	10	Explain how the study size was arrived at	Fig 1
Quantitative variables	11	Explain how quantitative variables were handled in the analyses. If applicable, describe which groupings were chosen and why	Methods/exposure status

	Item No	Recommendation	Page No
Statistical methods	12	(*a*) Describe all statistical methods, including those used to control for confounding	Methods/statistical analysis
		(*b*) Describe any methods used to examine subgroups and interactions	Methods/statistical analysis; Supplementary Information/results stratified by sex and calendar year
		(*c*) Explain how missing data were addressed	NA
		(*d*) *Cohort study*—If applicable, explain how loss to follow-up was addressed<br>*Case-control study*—If applicable, explain how matching of cases and controls was addressed<br>*Cross-sectional study*—If applicable, describe analytical methods taking account of sampling strategy	Methods ¶1 (matching is within individuals)
		(*e*) Describe any sensitivity analyses	Methods/statistical analysis; Supplementary Information/results of planned analysis
Results			
Participants	13*	(a) Report numbers of individuals at each stage of study—eg numbers potentially eligible, examined for eligibility, confirmed eligible, included in the study, completing follow-up, and analysed	Methods/study participants; Results/description of cases; Fig 1
		(b) Give reasons for non-participation at each stage	Methods/study participants; Fig 1
		(c) Consider use of a flow diagram	Fig 1
Descriptive data	14*	(a) Give characteristics of study participants (eg demographic, clinical, social) and information on exposures and potential confounders	Results/description of cases; Table 1
		(b) Indicate number of participants with missing data for each variable of interest	Table 1 (data were only missing for region, deprivation, and ethnicity, which were only use to describe the sample rather than in analysis)
		(c) *Cohort study*—Summarise follow-up time (eg, average and total amount)	NA (case-crossover study)
Outcome data	15*	*Cohort study*—Report numbers of outcome events or summary measures over time	NA (case-crossover study)
		Case-control study—Report numbers in each exposure category, or summary measures of exposure	NA (case-crossover study)
		Cross-sectional study—Report numbers of outcome events or summary measures	NA (case-crossover study)
Main results	16	(*a*) Give unadjusted estimates and, if applicable, confounder-adjusted estimates and their precision (eg, 95% confidence interval). Make clear which confounders were adjusted for and why they were included	Results/results of case-crossover analysis; Fig 3
		(*b*) Report category boundaries when continuous variables were categorized	Methods/exposure status (exposure boundaries, e.g. 1-2 days after hospital discharge)

	Item No	Recommendation	Page No
		(*c*) If relevant, consider translating estimates of relative risk into absolute risk for a meaningful time period	NA
Other analyses	17	Report other analyses done—eg analyses of subgroups and interactions, and sensitivity analyses	Results/results of case-crossover analysis ¶2; Supplementary Information/results of planned analysis; Supplementary Information/results stratified by sex and calendar year
Discussion			
Key results	18	Summarise key results with reference to study objectives	Discussion ¶1
Limitations	19	Discuss limitations of the study, taking into account sources of potential bias or imprecision. Discuss both direction and magnitude of any potential bias	Discussion/strengths and limitations ¶2-5
Interpretation	20	Give a cautious overall interpretation of results considering objectives, limitations, multiplicity of analyses, results from similar studies, and other relevant evidence	Discussion ¶1; Discussion/interpretation
Generalisability	21	Discuss the generalisability (external validity) of the study results	NA
Other information			
Funding	22	Give the source of funding and the role of the funders for the present study and, if applicable, for the original study on which the present article is based	Additional information/funding

S8. References for supplementary information

1 Morrow RL, Bassett K, Maclure M, *et al.* Outcomes associated with hospital admissions for accidental opioid overdose in British Columbia: a retrospective cohort study. *BMJ Open* 2019;**9**:e025567. doi:10.1136/bmjopen-2018-025567

2 NHS Digital. Hospital Admitted Patient Care Activity 2018-19. 2019.https://digital.nhs.uk/data-and-information/publications/statistical/hospital-admitted-patient-care-activity/2018-19 (accessed 5 Feb 2021).

3 Lewer D, Copeland C, Eastwood B, *et al.* Fatal opioid overdoses in healthcare settings in England: a case series analysis (PROTOCOL). 2020.https://doi.org/10.14324/000.rp.10116745

4 Whitaker HJ, Ghebremichael-Weldeselassie Y. Self-Controlled Case Series Methodology. *Annu Rev Stat Its Appl* 2019;**6**:241–61. doi:10.1146/annurev-statistics-030718-105108

5 Farrington CP, Whitaker HJ, Hocine MN. Case series analysis for censored, perturbed, or curtailed post-event exposures. *Biostatistics* 2008;**10**:3–16. doi:10.1093/biostatistics/kxn013

6 Schwarzer G. meta: An R Package for Meta-Analysis. *R News* 2007;**7**:40–5.
