# Supplementary material for: Fatal opioid overdoses during and shortly after hospital admissions in England: A case-crossover study
Source: PLoS Med. 2021 Oct 5;18(10):e1003759. doi: 10.1371/journal.pmed.1003759 (PMC8491890; doi:10.1371/journal.pmed.1003759)
Supplement: Attachment — Submitted filename: response_to_reviews_v4.pdf [file pmed.1003759.s002.pdf]

Re: "Fatal opioid overdoses during and shortly after hospital admissions in England: case-crossover study" (PMEDICINE-D-21-01225R1)

Response to reviews and editor requests

18 May 2021

University College London, Gower Street, London WC1E 6BT
Tel: +44 (0)20 7679 2000
email@ucl.ac.uk
www.ucl.ac.uk

Requests from editor

1. Abstract summary - At this stage, we ask that you reformat your non-technical Author Summary

We have included an Author Summary after the abstract.

2. Abstract: Please structure your abstract using the PLOS Medicine headings (Background, Methods and Findings, Conclusions). Please combine the Methods and Findings sections into one section, “Methods and findings”.

We have now used this format.

3. Please include the actual amounts or percentages of relevant outcomes, not just hazard ratios.

The absolute numbers and percentages of deaths in each exposure status are given in the second paragraph of the results (under ‘description of cases’) and at the bottom of table 1.

4. Please include the important dependent variables that are adjusted for in the analyses.

The dependent variable is opioid-related death and the main independent variable is the proximity to hospital admission and discharge. We believe that your question is relating to confounding. The case-crossover design eliminates all fixed/time-invariant confounders, and we have improved our discussion of time-varying confounding (please see comment #20, below).

5. Please quantify the main results (with p values in addition to 95% CI)

We have added p-values to the abstract and results text, as well as figure 3.

6. In the last sentence of the Abstract Methods and Findings section, please describe the main limitation(s) of the study's methodology.

We have added the limitation: “The main limitation of the method is that it does not control for time-varying health or drug use within individuals, and hospital admissions coinciding with high-risk periods may in part explain the results.”

7. Please ensure that the study is reported according to the STROBE guideline, and include the completed STROBE checklist as Supporting Information. Please add the following statement, or similar, to the Methods: "This study is reported as per the Strengthening the Reporting of Observational Studies in Epidemiology (STROBE) guideline (S1 Checklist)."

We have completed the checklist and uploaded it with our manuscript. We have also added this statement to our methods.

8. In the ethics statement in the manuscript, please provide additional information about the patient records used in your retrospective study. Specifically, please ensure that you have discussed whether all data were fully anonymized before you accessed them and/or whether the IRB or ethics committee waived the requirement for informed consent.

We have added the following information: “Data were anonymised before analysis, and personal identifiers such as name, address, or NHS Number, were not available to the research team.”

9. Your study is observational and therefore causality cannot be inferred. Please remove language that implies causality, such as “risk of” or “risk”. Refer to associations instead.

We have edited the document and tried to ensure that causality is not implied. In some places, we have used the word ‘risk’, but hopefully the observational design is clear and causality is not implied, e.g: “Days 15+ of hospital admission were associated with lower risk of opioid-related death”. We are very happy to edit the text further if you feel that we are implying causality too strongly.

10. Please include p-values in tables and main text. Please specify the statistical test used to derive the p values.

We have now included p-values in the abstract, results text, and in figure 3. P-values are reported directly from conditional logistic regression.

11. Please use the "Vancouver" style for reference formatting and see our website for other reference guidelines

We have updated the references to use the style described in the PLOS Medicine instructions. Please let us know if any further editing is required.

Reviewer #1

12. To my mind, the central issue here is to what extent the ICD-10 codes selected accurately identify deaths from opiate overdose, which is the focus of the paper. It would be nice to see some discussion of the codes chosen in the main text of the manuscript - e.g. why include codes relating to Parkinson's medications or anti rheumatic drugs? It would also be nice if the authors could include a breakdown of the codes assigned for the patients in the study - e.g. if the vast majority were X42 or Y42, I would find that more persuasive.

We used the standard Office for National Statistics definition of drug poisoning, i.e. where the underlying cause of death is recorded as ICD-10 code X40-X44, X60-X64, X85, or Y10-Y14.[1] An internationally recognised approach to coding drug-related deaths is to use one of these codes as the "underlying" cause of death and then to identify specific substances using "T" codes in secondary diagnostic positions.[2] We only included deaths that had an ICD-10 code indicating opioids, i.e. T40.0-T40.4 and T40.6. When people die after using opioids they have often taken several different drugs, and many different combinations of drugs and alcohol will be represented in our population, but all have taken opioids. Some are likely to have taken unusual drugs such as Parkinson's medication alongside opioids, but most will have died due to using heroin, methadone, benzodiazepines, alcohol, and other common CNS depressants. We have now included a table in supplementary information (table S1) showing the breakdown of ICD-10 codes. 75% have X42 as the underlying cause of death and 95% have X42, X62, X44, or Y12.

13. An alternative explanation for the pattern of deaths observed would be that they relate directly to the hospital admission - e.g. partially treated infection, mental health crisis, venous thromboembolism - but that when a young person with a history of opiate dependence is found dead in the community, perhaps surrounded by injecting paraphernalia, doctors completing the death certificate assume that the cause of death is an overdose. This alternative explanation would be consistent with the higher mortality seen following discharges against medical advice and, possibly, with the high mortality seen following longer admissions (presumably these were associated with more severe underlying illness). It would be nice to include a detailed discussion of this issue, as it is key to the conclusions we draw from the results. Do we know what mortality in the post discharge period looks like in people without a history of opiate dependence, particularly following admission with similar problems (bacteraemias, liver failure, schizophrenia, etc)?

We had not previously considered this explanation for our results and we discussed this issue in detail amongst the author group.

It is possible that some of the deaths in our study were not drug poisonings, but in fact related to a different disease for which the decedent was recently hospitalised. It is not possible to measure this directly using our data; and some cases may have been misclassified in this way.

An important reassuring feature of death registration in England is that coroners routinely investigate and register drug-related deaths. In 2019, 99.4% of deaths meeting the definition of 'drug-related deaths' used in our study were certified by coroners.[1] This means that deaths in our study have undergone thorough investigation, including toxicological tests. The ONS then performs additional validation of drug-related deaths, with manual coding of the underlying cause of death based on notes and causes reported by coroners. This should be a further safeguard against misclassification.

We have added the following text to the methods section: "Coroners investigate drug-related deaths in England, including analysis of toxicology results. This means that the causes of death in this study have been validated to a greater degree than most deaths."

14. It would be good to include greater justification of some of the analytical choices made - e.g. why only look at 5 control days? Why 14 days post discharge as the period of interest?

Case-crossover designs measure the acute "triggering" effect of a transient exposure. The duration of exposure periods should reflect the time over which we expect this effect to occur.[3] The difference the "true" timing of the effect and the duration of exposure periods will dilute the associations observed in the study. Our considerations in choosing the exposure periods were: (1) to have a manageable number of exposure periods, (2) to capture the short-term risk associated with using drugs immediately after leaving hospital, i.e. days 1-2 after discharge, (3) that other studies suggest the risk of drug-related deaths is high in the 14 days after release from prison[4] and cessation of opioid agonist therapy[5], and similar mechanisms may act after hospital discharge.

15. The issues regards data availability are problematic but, I suspect, outside the authors' control.

That is correct; we are unable to share our own extract of the data due to the conditions of our access. However, the data are available to researchers who have appropriate permissions and we have added the following information to the data sharing statement: "Researchers can use the linked Hospital Episode Statistics and ONS mortality data used in this article via the NHS Digital Data Request Service, with more information at https://digital.nhs.uk/services/data-access-request-service-dars."

16. In the introduction, it would be good to include some justification for focusing on 'opioid related deaths' as opposed to 'opiate overdoses'. I note these terms are used slightly interchangeably in the manuscript. I would also like to see some statement about the frequency of these deaths in the population.

There is varying terminology for drug-related deaths. We felt that "opioid-related deaths" is most accurate because we are studying deaths due to drug poisoning where an opioid contributed to the death. It is usually not possible to determine which substance primarily caused the death.

We agree that the language should be consistent and have ensured that we refer to cases as "opioid-related deaths" throughout, rather than "overdose". We prefer "opioid-related deaths" because some people who die after using drugs such as heroin appear to have non-toxic concentrations of opioids, and poly-drug use is likely to be an important factor in many deaths.[6,7] We have retained the word "overdose" in the study title (which is now "Fatal opioid overdoses during and shortly after hospital admissions in England: case-crossover study") and in the non-technical author summary. This is because we were concerned that "opioid-related deaths" in the absence of more detailed case definition would make the reader assume that the article is about iatrogenic opioids. We have included the following text in the methods section to explain this terminology: "For simplicity, we refer to these deaths as "fatal opioid overdoses" in the title and author summary, though we use "opioid-related deaths" elsewhere to reflect the difficulty of attributing deaths to one specific drug."

We also refer you comment #37, below.

17. Inclusion in the study was conditional on having ever been admitted to hospital. It is good that the number of deaths excluded are quantified. It would be good to include some discussion of whether this may result in bias. I cannot immediately see that this would set up, e.g., collider bias. And I am not sure, anyway, whether that would be an issue in a case only analysis?

We do not think that exclusion of individuals never admitted to hospital would cause bias in terms of the relative association between hospital admission/discharge and opioid-related death. The excluded individuals were not admitted to hospital, and therefore it is not possible to infer the association between hospital admission and opioid-related death from these individuals. From a statistical point of view, the conditional regression only uses information from participants/strata where the exposure status varies between the day of death and at least one of the control days. Therefore, exclusion of people never admitted to hospital does not affect the odds ratios.

The exclusion of these individuals does change the interpretation of the absolute proportion of opioid-related deaths that occur during the exposure periods used in our study. We reported that 1088/13609 (8.0%) occurred in the 14 days after discharge. However, this denominator only includes people who appear in our hospital records. We estimated that 1959/16586 = 11.8% of opioid-related decedents are not in these hospital records; so we can estimate that 13609 * 1.118 = 15216 deaths would be eligible for inclusion in our study. Therefore, we can estimate that 1088/15216 = 7.1% of deaths (or 1 in 14) occur during this period. We have updated the text to reflect this.

18. The authors have deviated significantly from the analysis planned in the published protocol. The discussion of the reasons for this in supplementary materials is good. However, this deviation from the published protocol should be mentioned more prominently in the main text of the manuscript. Will the analysis of the NPSAD data be presented separately?

The main deviation from the protocol was the use a case-crossover method rather than a self-controlled case series. We have included the following text in the methods: "The protocol included a self-controlled case series analysis, and we chose a case-crossover design instead because it allowed analysis of exposures with varying durations."

We are doing the analysis of NPSAD alongside this study. As it uses a different dataset and method, we decided to publish the results separately to keep this article focused.

19. There is good discussion about discrepant documentation of dates of discharge and death. However, these discordant dates do raise questions about the accuracy of the dates coded in the two databases - errors won't always be so obvious. Do we know anything about how accurate these data are? If so, some comment on this would be valuable, given accurate dates are key to the analysis presented.

We agree that large and obvious discrepancies in some individuals may seem to suggest smaller and less obvious discrepancies/errors in other individuals. However, there is no obvious reason why dates would be "wrong", and there are good reasons for small discrepancies between death dates recorded by ONS and discharge dates recorded by hospital where the patient died. The two reasons are (1) if the death occurred around midnight; and (2) if the hospital discharged a patient after death to allow for post-mortem investigations or organ donations. In both scenarios the date of death recorded in ONS data is likely to be before the final discharge date in Hospital Episode Statistics, which was borne-out when we compared the dates in our raw database extract. The large discrepancies in a small number of individuals are likely due to failed linkage rather than inaccurate recording of dates, and we have added the following information to the methods: "These discrepant dates are likely due to failed linkage rather than inaccuracies in hospital discharge or death dates".

20. A clear statement about whether any attempt was made to adjust for time varying confounders is needed.

We adjusted for weekday by selecting control days with the same weekday as the day of death, and this is described on page 7 (methods). We expected that the weekday would be a more important confounder than season.

Other important confounders are those that relate to drug use and health, which are not measured. We tried to limit this type of confounding by selecting control days no more than two years prior to the event. We have strengthened our discussion of this limitation and added the following text to the abstract: "The main limitation of the method is that it does not control for time-varying health or drug use within individuals, and hospital admissions coinciding with high-risk periods may in part explain the results" (as PLOS Medicine requires the key methodological limitation to be stated in the abstract).

The other potential time-varying confounder is age, which is measured. The case-crossover method does allow for adjustment by age, but only if the control days are both before and after the case/event day. In a design

such as ours, the control days are always before the case day and therefore the event always occurs at the maximum age within individuals, meaning that the association between age and the event is difficult to estimate. We believe this is a minor problem because (1) existing evidence suggests that the risk of drug-related death is not strongly associated with age, unlike the risk of death due to diseases such as cardiovascular disease and cancer, which increases rapidly with age among people who use illicit opioids;[8] (2) control days are a maximum of two years before deaths, so age can only vary by two years or less; (3) the self-controlled case series model for event-dependent exposures[9] (reported in Supplementary Information) does allow for adjustment by age, and inclusion of age in this model did not change the results.

21. I think 'Died in hospital following admission with an overdose' (or similar) (as in the tables) is clearer than 'occurred in hospital following drug use in the community' (as used in the text). It seems likely that many/most of the cohort were using drugs in the community.

We agree and have edited the text to this effect – though we have used "died in hospital following admission due to drug poisoning" (see comment #16 re: use of the word "overdose").

22. Do we know anything about how accurately discharge against medical advice is coded in HES? Note, this variable is not always binary - sometimes clinicians try to accommodate patients' preferences for a shorter admission, where they would have preferred a longer one. Such instances won't be coded as 'discharge against medical advice'.

We agree that the binary variable "discharge against medical advice" is a crude classification of a range of different situations. We are not aware of any more detailed description of this variable – there is nothing in the Hospital Episode Statistics data dictionary.[10] We emailed NHS Digital (the central body that collates hospital data) and they were not aware of any guidance that hospitals use when classifying discharge types. We also discussed this with some doctors currently working in hospital wards in England, who said that it is simply a tick-box in the discharge forms, i.e. the interpretation of "discharge against medical advice" is up to clinical teams who record this information.

If a patient decides to leave before treatment is completed, but are still discharged normally, this might be considered a misclassified admission. This is non-random misclassification and the direction of effect on our results is difficult to estimate. We have added to the methods to clarify the source of this variable, and also discussed the limitations in the recording of this variable: "Our use of a national hospital dataset meant that we were able to include all hospital admissions in England, but it also meant that we had limited detail about individual patients. One limitation relates to 'discharge against medical advice'. This was a binary variable in our analysis, when in reality there are a range of scenarios where discharge is negotiated between patients and hospital staff."

23. The paragraph at the end of page 9, which finishes on page 10, is not clearly written. An attempt is made to describe, in a single sentence, the reasons for hospital admission both overall, and in those who died following overdose in hospital. The sentence ends up being very long, with lots of semi colons. This should be rewritten.

The purpose of providing information about hospital admissions prior to death was to help contextualise the relative effects reported in the main analysis. For example, we can see that discharge against medical advice is associated with opioid-related death, so it is useful to know that 10% of admissions ended with discharge against medical advice. We have retained this information. The other information related to the frequency of admission for non-fatal drug poisoning and alcohol use, and the causes of admission for patients who died in hospital. This information seems interesting but of secondary importance. Given the complexity and post-hoc nature of analysing the hospital diagnoses, we decided to exclude this section of the results.

24. The relative risk of death during days 1-14 of hospital admission in the abstract differs to the relative risk presented in the results section. I would give the figures for the relative risk of fatal overdose on days 15+ of hospital admission in the main text of the manuscript (results section). I think the relative protection from overdose provided by a psychiatric admission, or by a medical admission of longer duration, is worth commenting on.

There are plausible mechanisms for the days 15+ being lower risk. For example, there are sometimes delays to prescription of opioid agonist therapy in hospital, and those staying for longer may be more likely to receive it. However, we were cautious about interpreting this finding due to possible selection bias. Admissions are of varying length, and patients admitted involuntarily or those who are able to tolerate a longer hospital stay (with less severe withdrawal, or better-controlled pain) may be at less risk of overdose for these reasons. We have added this limitation to the discussion. We do not know why psychiatric admissions appear to be associated with lower risk of opioid-related death. Our best theory is that involuntary admissions make it more difficult to acquire drugs or leave a ward temporarily to use drugs, but we decided not to speculate in the manuscript.

25. Paragraph at the end of page 12 - this is good. It might be worth explicitly discussing pain here. Many conditions that warrant admission to hospital are painful, and therefore the association between overdose and admission to hospital may be driven by increased opiate consumption (both illicit and prescribed) to manage associated pain. Pain could also be discussed in the relevant paragraph on page 13 (immediately after the 'Interpretation' header).

We agree and have added a discussion of pain in the first paragraph under ‘Interpretation’.

26. The protocol describes a planned analysis looking at risk of death during the 14 days prior to hospital admission ('period Z'). I assume this was not done because it is not possible - you cannot know whether people who die are in period Z, because people are not admitted to hospital post mortem. Some comment about this planned analysis would be valuable. The planned disaggregation of the overdose deaths into accidental and intentional, which I think is possible, would be good to see.

You are correct about ‘period Z’ and we have included a comment in the Supplementary Information explaining why this exposure status was not included in the analysis.

You are also correct that disaggregation of deaths into accidental and intentional is also possible, and based on ICD-10 codes 1,217/13,609 deaths (8.9%) in our study were intentional, where intentional drug poisonings are those with an underlying cause of ICD-10 X60:64. For your interest, we have included this disaggregation below. The association between opioid-related suicides and recent hospital discharge appears smaller than the association between accidental opioid-related deaths and hospital discharges, which may suggest that accidental overdose is the main risk after discharge. However, we decided not to include the disaggregation in the article because intentionality is difficult to determine and therefore this stratification is difficult to interpret.

Table: Main results stratified by recorded intention. Values are conditional odds ratios (95% confidence intervals) with a reference of time spent in the community

	Suicides (those with an underlying cause of ICD-10 X60:X64)	All other opioid-related deaths
A: Days 1-14 of admission	0.79 (0.45-1.37)	1.05 (0.89-1.25)
B: Days 15+ of admission	0.82 (0.37-1.82)	0.37 (0.26-0.52)
C: Days 1-2 after discharge	2.21 (1.20-4.08)	4.63 (3.93-5.46)
D: Days 3-14 after discharge	1.62 (1.19-2.21)	2.14 (1.95-2.33)

27. The limitations section in the protocol is better than the limitations section in the manuscript - much of the discussion from the protocol should be included in the manuscript.

We have now added to the limitation section, as per other comments. We also revisited the limitations in the protocol: (1) **'Missing deaths'** – this is addressed in the methods section, but we decided not to extend the limitations section because this is a minor limitation for a case-only analysis (as flagged in the protocol – also see comment #17); (2) **'Misclassification of hospital admissions ending in death according to overdose vs.other causes'**. We agree that this limitation should be highlighted in the manuscript and have added: "A second limitation relates to our classification of hospital admissions ending in opioid-related death. If an admission ended in opioid-related death and the primary cause of admission was a common complication of opioid poisoning, such as cardiac arrest, then we assumed that opioid poisoning was the indication for admission. This is a sensitive approach, and in some cases the opioid poisoning may have happened while the patient was in hospital. Therefore, it is possible that we have underestimated the association between hospital admissions (exposure periods A and B) and opioid-related death." (3) '**Limitations related to the case definition.'** Hopefully this is now covered more explicitly in Supplementary Information (table 1). Please also see comments #12 and #37. (4) '**The design estimates relative effects only'**. This is a limitation of the method but we did not feel it required specific discussion in the manuscript. (5) **'It may be difficult to evaluate intent'**. For this reason, we decided not to include any breakdown or analysis of intent/suicide (please also see comment #26. (6) **'Some time-varying confounders will not be measurable'**. This issue is already discussed in the limitations (please also see comment #20).

Please let us know if you think any of these (or other) limitations should be discussed more prominently.

28. The figure on page 6 of supplementary materials is unreadable. I think this should be split into a number of separate figures. Note, the reference to this figure in the text on the previous page is missing a number.

We have simplified this figure by reducing the number of sensitivity analyses (see comment #44). We have also made the figure easier to read by rotating it, using a vector format (meaning you can zoom into the PDF without the figure pixelating), and including a table of results below the figure. Let us know if you think the figure could be improved further.

29. Only two authors seem to have made financial disclosures. Who paid for this work? If the other authors have no disclosures, that should be stated.

We have included all relevant funding and competing interests. We have added "Other authors declare no competing interests."

30. When the manuscript is resubmitted, please include line numbers, as this makes things easier for reviewers!

We have added line numbers.

31. In Figure 2, it would be good to define the dotted lines in the legend. I would also say 'control day', rather than 'control period'.

We have made various improvements to figure 2, including defining the dotted line and using the term 'control day' (which we agree is better).

32. To my mind, the opposite of acute is elective, and the opposite of psychiatric is non-psychiatric. I suggest 'non-psychiatric' would be a better term than 'acute', given there might have been some planned admissions.

We agree and have edited the text and figure 3 to use the term 'non-psychiatric' instead of 'acute'.

33. In the first paragraph after the 'Interpretation' header, should it read 'more vulnerable to death following an opioid overdose'?

Given the issues with the term ‘overdose’ (see comment #16), we prefer to say “death after using opioids”, but please let us know if you feel this wording is unclear.

34. Is it worth discussing patient and peer BLS training, in addition to naloxone provision, as potential interventions to prevent death from overdose?

We agree, and have edited the discussion to include the potential benefit of basic life support training (including the acknowledgement this this, as well as naloxone, are most likely to benefit peers rather than the patient themselves).

35. I agree with the final paragraph, but it seems an odd way to conclude the manuscript.

We have now added a ‘conclusions’ subheading at the end of the discussion, and used the text from the ‘conclusions’ section of the abstract.

University College London, Gower Street, London WC1E 6BT
Tel: +44 (0)20 7679 2000
email@ucl.ac.uk
www.ucl.ac.uk

Reviewer #2

36. P2. Findings - 1.7%, not clear what the denominator is (of total deaths, or total exposures)
The 'similar or lower' should be qualified to the different periods - a bit confusing otherwise.

We have now included denominators wherever a proportion is quoted, and hopefully this is now clear. We have also added the odds ratios for the hospital admission (exposure periods A and B) to the abstract.

37. P4. Study participants. Do the external cause and ICD-10 codes distinguish between iatrogenic opioid poisonings and others? Presumably medication error could lead to an adverse drug event (particularly in someone vulnerable e.g. with poor kidney or hepatic function). Is the external cause data available specific to agents (e.g. prescription vs. non-prescription opioid forms)? There is some discussion of this in the protocol but not in the main manuscript or supplementary appendix.

The ICD-10 codes do not easily distinguish between iatrogenic and illicit opioid poisoning. Toxicology cannot usually distinguish between illicit and prescribed opioids – for example heroin and morphine are not distinguishable. As a result, opioid-related deaths are sometimes coded as ‘T40.2: other opioids’ or ‘T40.6: other and unspecified narcotics’ (see new table S1 in supplementary information) when the exact opioid is unknown. Sometimes there is other evidence that coroners can use to record use of illicit drugs, such as the presence of drugs at the scene of death, and some deaths are assigned the code ‘T40.1: heroin’. We have done a post-hoc subanalysis of the 3,953 deaths with this code and found similar associations to our main results (see table below).

Another important consideration is the demographic profile of decedents, which is mostly young males living in deprived areas (table 1 in the main manuscript). This reflects the characteristics of people in treatment for opioid dependence[11] and people who inject drugs[12] in England. In contrast, patients who are prescribed opioids are mostly older and the majority are female.[13]

These factors suggest that the opioid-related deaths in our study are primarily associated with illicit opioids, though many participants may also use prescribed opioids either for opioid agonist therapy or pain relief. Our forthcoming analysis of NPSAD (described in our protocol) will help us understand the potential role of prescribed opioids in the high risk of opioid-related death post-discharge, as this source includes records of opioid prescriptions.

Table: main results stratified by contribution of heroin to death. Values are conditional odds ratios (95% confidence intervals) with a reference of time spent in the community

	Deaths where heroin (ICD-10 T40.1) contributed to death	All other opioid-related deaths
A: Days 1-14 of admission	0.86 (0.61-1.23)	1.08 (0.90-1.30)
B: Days 15+ of admission	0.29 (0.15-0.56)	0.47 (0.32-0.67)
C: Days 1-2 after discharge	5.84 (4.30-7.94)	3.95 (3.28-4.75)
D: Days 3-14 after discharge	2.16 (1.83-2.56)	2.07 (1.87-2.28)

38. P5. Was there any evidence for time-trends, i.e. overall changes in opioid deaths in hospitals? If there were this would presumably bias the results towards a positive association.

Our protocol did not include an analysis of time trends, partly because we did not have a hypothesis that the association between hospital admission and opioid-related death would change over time. As a post-hoc analysis, we have stratified the results by calendar year of death and included the results in Supplementary Information. Although it is difficult to test for time trends directly using our method, these results do not suggest any important changes in the relative association between hospital admission and opioid-related death.

39. P6. Figure 2. Nice figure - my only quibble is that the diamond marker can overlap into a hospital admission period on the figure even where it occurs outside the period. Perhaps a line or other marker instead?

Thanks for this feedback. We have made various improvements to this figure, including a reduction in the size of the diamond so it no longer obscures the end of the hospital admission period.

University College London, Gower Street, London WC1E 6BT
Tel: +44 (0)20 7679 2000
email@ucl.ac.uk
www.ucl.ac.uk

Reviewer #3

40. Introduction: add references for statements "Sometimes staff are too busy to verify a patient's usual dose of methadone or buprenorphine, or do not have sufficient knowledge or training about opioid dependence" and "Opioid withdrawal can lead patients to leave hospital to buy drugs. Some bring a supply into hospital to keep them going, and some arrange for dealers to visit them while they are staying on a ward."

We have now included references for these statements.

41. Methods, exposure status: Consider adding clear language stating that the sample included patients who had an opioid-related death but who were admitted for causes other than opioid use.

Another reviewer also felt this language could be clearer (see comment #21), and where we refer to these cases we have updated the language to “died in hospital following admission due to drug poisoning”. Please let us know if any parts of the text remain unclear.

42. Methods, statistical analysis: Consider adding more information about the approach used for the analysis, why it was selected as the best tool to analyze the data.

We have added the following justification of the design to the methods section: “We chose this design because it allows inclusion of a large proportion of cases and is statistically powerful, and it controls confounding more effectively than a cohort design that might compare people who use opioids and are admitted to hospital with those not admitted.”

43. Discussion: results by gender have some interesting findings (currently in the appendix); consider including this stratified result by gender in the results and discussion.

We agree that the results for men and women are interesting, because existing evidence shows that men and women face different risk environments for overdose. This stratification was also specified in our protocol. The results suggest a similar pattern for men and women. We have therefore signposted this analysis in both the methods and results sections of the article.

Reviewer #4

44. Confounding by escalating drug use/deteriorating health over time: The authors appropriately note that their findings may be partially be explained by escalating drug use or deteriorating health over time. This is a strong limitation. I wonder if they could do a sensitivity analysis extending the control window back just 1 year instead of 2, and see if their results are similar (is this what was done in appendix 4? It's not totally clear to me. If so, would move these results regarding sensitivity analysis with varying time windows into the main manuscript). This might give some understanding of the degree to which this type of confounding could have influenced their results.

We agree that this is a key limitation and we have now highlighted this limitation in the abstract, as PLOS Medicine requires the key methodological limitation to be given in the abstract. You are correct that the original appendix included some sensitivity analysis of the control days, though the approach was slightly different to the main analysis. The original sensitivity analyses used control days at fixed periods before death, while the main analysis sampled control days from a period before death. We have therefore followed your suggestion and implemented a sensitivity analysis that is more consistent with the main analysis, sampling control days from 1 year before death (rather than 2 years). We also did a second sensitivity analysis sampling control days from 3 years before death. The results are given in Supplementary Information. The direction of difference from the main analysis is as-expected: when control days are sampled from 1 year before death the association between hospital admission and opioid-related death is reduced, and when control days are sampled form 3 years before death the association is increased. This suggests that time-varying confounding such as escalating drug use/deteriorating health within individuals may in part explain the results, though the sensitivity analyses do not differ substantially from the main results.

We also note that the policy or clinical relevance of the results may be similar if the results are explained by this type of time-varying confounding. While this would mean that hospital admissions did not cause opioid-related deaths (i.e. the same number of deaths would have happened in the absence of hospital admissions), the admissions are still markers of risk and therefore opportunities for intervention.

45. Possible selection bias: The authors state, "This database does not include deaths if no linkage is found (most likely because the decedent was never admitted to hospital)." This seems like it would tend to bias towards inclusion of individuals with a history of hospitalization, which could potentially increase the association between hospitalization and death, since individuals are also selected for inclusion by virtue of having experienced opioid-related death. If I am understanding the database correctly, this should be added as a limitation.

Please see comment #17. Our data should include all opioid-related deaths where the decedent also appears in the national database of hospital episodes at any time. We do not think that the exclusion of decedents who were never hospitalised will lead to bias in the relative association between hospital admission/discharge and opioid-related death. However, it does affect our estimate of the proportion of opioid-related deaths in the population that occur during exposure periods, and we have edited the text to reflect this.

46. Abstract: The abstract starts by stating that some hospital patients use illicit drugs while admitted, and there have been reports of patients found dead on hospital premises after using illicit drugs such as heroin. This led me to believe this was a study about use of opioids in hospitals, which is not a main focus of the analysis. I would instead start by stating why the time just after hospital discharge may be a time of heightened risk.

We have edited this part of the abstract to make it clear that the article focuses on both the admission itself and the period after discharge: “Hospital patients who use illicit opioids such as heroin may use drugs during an admission, or experience withdrawal and leave hospital to use drugs. There have been reports of patients

found dead from drug poisoning on hospital premises or shortly after leaving hospital. This study examines whether hospital admission and discharge are associated with increased risk of opioid-related death.”

University College London, Gower Street, London WC1E 6BT
Tel: +44 (0)20 7679 2000
email@ucl.ac.uk
www.ucl.ac.uk

References for response letter

1. Office for National Statistics. Deaths related to drug poisoning in England and Wales QMI. 2018. Available: https://www.ons.gov.uk/peoplepopulationandcommunity/birthsdeathsandmarriages/deaths/methodologies/deathsrelatedtodrugpoisoninginenglandandwalesqmi
2. EMCDDA. EMCDDA standard protocol to collect data and report figures for the key indicator drug-related deaths (DRD-Standard, version 3.2). European Monitoring Centre for Drugs and Drug Addiction; 2009. Available: https://www.emcdda.europa.eu/html.cfm/index107404EN.html_en
3. Maclure M. The Case-Crossover Design: A Method for Studying Transient Effects on the Risk of Acute Events. Am J Epidemiol. 1991;133: 144–153. doi:10.1093/oxfordjournals.aje.a115853
4. Binswanger IA, Heagerty PJ, Koepsell TD. Release from Prison — A High Risk of Death for Former Inmates. N Engl J Med. 2007; 9.
5. Sordo L, Barrio G, Bravo MJ, Indave BI, Degenhardt L, Wiessing L, et al. Mortality risk during and after opioid substitution treatment: systematic review and meta-analysis of cohort studies. BMJ. 2017;357. doi:10.1136/bmj.j1550
6. Darke' S, Zador D. Fatal heroin 'overdose': a review. Addiction. 1996;91: 1765–1772. doi:10.1046/j.1360-0443.1996.911217652.x
7. Druid H, Strandberg JJ, Alkass K, Nyström I, Kugelberg FC, Kronstrand R. Evaluation of the role of abstinence in heroin overdose deaths using segmental hair analysis. Forensic Sci Int. 2007;168: 223–226. doi:10.1016/j.forsciint.2006.02.047
8. Degenhardt L, Larney S, Randall D, Burns L, Hall W. Causes of death in a cohort treated for opioid dependence between 1985 and 2005: Mortality among opioid-dependent people. Addiction. 2014;109: 90–99. doi:10.1111/add.12337
9. Farrington CP, Whitaker HJ, Hocine MN. Case series analysis for censored, perturbed, or curtailed post-event exposures. Biostatistics. 2008;10: 3–16. doi:10.1093/biostatistics/kxn013
10. NHS Digital. HES Data Dictionary: Admitted Patient Care. 2020. Available: https://digital.nhs.uk/data-and-information/data-tools-and-services/data-services/hospital-episode-statistics/hospital-episode-statistics-data-dictionary
11. Public Health England. Substance misuse treatment for adults: statistics 2019 to 2020. Public Health England; 2018. Available: https://www.gov.uk/government/statistics/substance-misuse-treatment-for-adults-statistics-2019-to-2020
12. Public Health England. Unlinked Anonymous Monitoring (UAM) Survey of HIV and viral hepatitis among PWID: 2020 report. Public Health England; 2020. Available: https://www.gov.uk/government/publications/people-who-inject-drugs-hiv-and-viral-hepatitis-monitoring
13. Jani M, Birlie Yimer B, Sheppard T, Lunt M, Dixon WG. Time trends and prescribing patterns of opioid drugs in UK primary care patients with non-cancer pain: A retrospective cohort study. Song Z, editor. PLOS Med. 2020;17: e1003270. doi:10.1371/journal.pmed.1003270
