# Supplementary material for: Fatal opioid overdoses during and shortly after hospital admissions in England: A case-crossover study
Source: PLoS Med. 2021 Oct 5;18(10):e1003759. doi: 10.1371/journal.pmed.1003759 (PMC8491890; doi:10.1371/journal.pmed.1003759)
Supplement: Attachment — Submitted filename: response_to_reviews_v5.pdf [file pmed.1003759.s003.pdf]

Re: "Fatal opioid overdoses during and shortly after hospital admissions in England: case-crossover study" (PMEDICINE-D-21-01225R2)

Response to reviews and editor requests

28 June 2021

University College London, Gower Street, London WC1E 6BT
Tel: +44 (0)20 7679 2000
email@ucl.ac.uk
www.ucl.ac.uk

Requests from editor

1. Thank you for providing your STROBE checklist. Please replace the page numbers with paragraph numbers per section (e.g. "Methods, paragraph 1"), since the page numbers of the final published paper may be different from the page numbers in the current manuscript.

We have updated the STROBE checklist.

2. If possible, please include a summary of ethnic/ racial characteristics in table 1.

Death records in the UK do not include the ethnicity/race of the decedent. Hospital records do include information about ethnicity. There are known issues with this data, including missingness, inconsistency between records, and validity when compared to self-reported ethnicity.[1,2] We used the following algorithm to determine ethnicity from hospital data: (1) use the most frequently recorded ethnicity, excluding ‘unknown’; (2) if two or more ethnicities excluding ‘unknown’ are recorded an equal number of times, use the most recently recorded ethnicity from the tied categories; (3) use ‘unknown’ if this is the only recorded value. We have also included a brief caption/note on Table 1 to explain that ethnicity information is derived from hospital data.

91% of decedents were recorded as having ‘White British’, ‘White Irish’, or ‘White Other’ ethnicity. This is similar to people in treatment for opiate dependence in England in 2019. In this population, 88% have ‘White’ ethnicity.[3]

We have also corrected the row labels in Table 1.

3. Please ensure that all weblinks are current and accessible. For example, the weblink for references #8, 9, 14, are broken

We have checked all the weblinks and they do all seem to be working, including 8, 9, and 14, so perhaps these were temporarily unavailable when you tested them. Please let us know if you continue to have problems. For links to newspaper articles, we have used the ‘archive.org’ service as we have found this preserves the content more sustainably, though it can be slow to load at times.

University College London, Gower Street, London WC1E 6BT
Tel: +44 (0)20 7679 2000
email@ucl.ac.uk
www.ucl.ac.uk

Reviewer #1

4. My main residual concern relates to the authors' response in point 13 - I am not fully persuaded. I write death certificates and liaise with coroners when on clinical duty. In hospitalised patients, where deaths are sudden and unwitnessed, it is sometimes hard to attribute a cause of death. Where someone is found dead in the community - with no access to regular patient observations, blood test results, etc - I imagine things are even less certain.

   I am not convinced that toxicology, or a coroner reviewing what may be limited information, really helps here. Finding opiates in somebody who is opiate dependent does not tell us whether their death was a result of accidental poisoning (X42, etc), a result of the condition that recently put them into hospital, or a result of complications associated with that hospitalisation (venous thromoembolism, etc).

   I would like to see greater acknowledgement of the inherent difficulty in attributing cause of death. If data are available on the proportion of these deaths that were witnessed, and the proportion of cases that underwent post mortem, that should be presented. I would like to see mention of misattribution of cause of death as a possible alternative explanation for the observed mortality patterns in the discussion.

Thank you for pursing this issue and we do agree that the study is strengthened by ensuring this limitation is clear. We have added a paragraph to the discussion (pages 14-15; lines 301-307), including some paraphrased text from your feedback. We hope that this is clear but please let us know if you feel it can be improved further.

5. In table 2, I struggle to see how the median can be 1, where only 40% of the observations are 1? This may be a rounding issue.

Thank you for pointing this out. This was a mistake in the table. Elsewhere in the study, we have considered an admission where discharge occurred on the same day as admission as having duration of 1 day, and 2 days if discharge was on the following day. When we initially calculated the median and mean durations, we counted these scenarios as 0 and 1 days respectively. This is now corrected.

University College London, Gower Street, London WC1E 6BT
Tel: +44 (0)20 7679 2000
email@ucl.ac.uk
www.ucl.ac.uk

Reviewer #3

6. The use of the word "proximity" initially I understood it as a space measure, which is not the focus of this paper. It may be worth adding an adjective to clarify the intended meaning, i.e., "time proximity."

We agree that this makes the language clearer, and have edited the text.

7. As the use of illicit, more potent, and fatal opioids (mainly fentanyl) increases in our populations, I wonder whether the estimates have a significant upward time trend (i.e., risk of death in the two weeks after hospital discharge was higher for patients admitted in 2018-2019 than for patients admitted in 2010-2011).

To our knowledge, there are to-date limited synthetic opioids in the illicit drugs market in the UK. In our revised manuscript we added a post-hoc analysis stratified by calendar year (see Supplementary Information, 'results stratified by sex and calendar year'), and did not observe any obvious change over time in the relative risk associated with hospital admission or discharge. This may change in the future if synthetic opioids become more common in the UK.

References for response letter

1. Saunders CL, Abel GA, El Turabi A, Ahmed F, Lyratzopoulos G. Accuracy of routinely recorded ethnic group information compared with self-reported ethnicity: evidence from the English Cancer Patient Experience survey. BMJ Open. 2013;3: e002882. doi:10.1136/bmjopen-2013-002882
2. Mathur R, Bhaskaran K, Chaturvedi N, Leon DA, vanStaa T, Grundy E, et al. Completeness and usability of ethnicity data in UK-based primary care and hospital databases. J Public Health. 2014;36: 684–692. doi:10.1093/pubmed/fdt116
3. Public Health England. Substance misuse treatment for adults: statistics 2019 to 2020. Public Health England; 2018. Available: https://www.gov.uk/government/statistics/substance-misuse-treatment-for-adults-statistics-2019-to-2020

University College London, Gower Street, London WC1E 6BT
Tel: +44 (0)20 7679 2000
email@ucl.ac.uk
www.ucl.ac.uk
